# Valorization of Brewer’s Spent Grain Through Ultrasound-Assisted Extraction of Phenolic Compounds Using Deep Eutectic Solvents

**DOI:** 10.1021/acsomega.4c10526

**Published:** 2025-05-29

**Authors:** Paloma Paiva Santiago, Fabiano André Narciso Fernandes, Rílvia Saraiva de Santiago-Aguiar

**Affiliations:** † Energy, Environmental and Chemical Engineering (EECE), McKelvey School of Engineering, 7548 Washington University in St. Louis (WashU), St. Louis, Missouri 63130-4899, United States; ‡ Chemical Engineering Department, 28121 Federal University of Ceara, Campus do Pici, Bloco 709, 60440-900 Fortaleza, CE, Brazil

## Abstract

Brewer’s Spent Grain (BSG) is a residue produced in large quantities of breweries. BSGs have a high nutritional value and are a source of bioactive compounds, suggesting that this residue can be reused to produce high-value products with industrial applications. Among the bioactive compounds, the BSGs have high concentrations of phenolic compounds that are in high demand in the cosmetic and food industry. In this work, an ultrasound-assisted extraction using deep eutectic solvents (DESs) was evaluated to recover phenolic compounds from BSG. A screening of four DESs and their mixtures with 25% water (v/v) identified DES choline chloride and oxalic acid (ChCl:OA) and DES choline chloride and lactic acid (ChCl:LA) as the most effective solvents, achieving phenolic compound yields of 0.201 and 0.172 mg GAE/L, respectively, during the initial screening. To further enhance the extraction efficiency, a two-step serial extraction process was employed. The first step used hexane or ethanol to remove oils and sugars, which reduced the impurities and increased the selectivity of DES in the second step. The highest phenolic compound yields were obtained with ethanol-ChCl:OA and hexane-ChCl:OA systems, achieving 0.513 and 0.516 mg GAE/L, respectively, at 30 °C. Notably, ChCl:OA, the most viscous DES among those tested, demonstrated the highest extraction efficiency, suggesting that its chemical composition and interactions with the BSG matrix may have compensated for its higher viscosity. Serial ultrasound-assisted extraction effectively removed oils and sugars from the BSG sample and increased the efficiency of the DES in recovering phenolic compounds.

## Graphical abstract

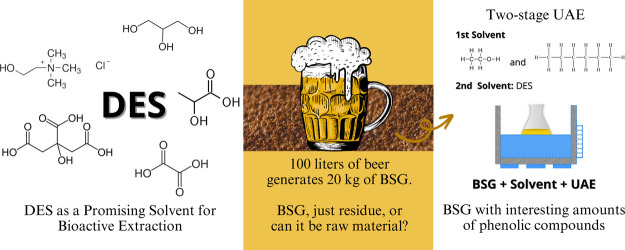

## 1 Introduction

The beer market is growing steadily, with an expected production of 189.3 billion L by 2027. China, the United States, and Brazil are the largest beer producers.^1^ Beer processing generates a large amount of Brewer’s Spent Grain (BSG), which is considered an industrial residue. It is estimated that for every 100 L of beer, 20 kg of BSG is produced.^2^ In 2022 alone, global beer production totaled 1.89 billion hectoliters, generating more than 3.7 million tons of BSG.^3^

The environmentally correct destination for BSG is its disposal in a landfill or composting, where it will decompose, producing other byproducts, such as methane and leachate. BSG can also be used as supplemental feed for livestock since it contains sugars, fatty acids, protein, and other minor compounds. BSG is a material rich in bioactive compounds; therefore, reusing this residue as a raw material in other industrial processes can be an interesting alternative to reducing the environmental impact caused by its disposal while obtaining high-value bioactive compounds.

BSG contains 57 types of flavonoids, 26 phenols and 21 other bioactive compounds, making it a good source of raw material for the extraction of phenolics and other bioactive compounds that are applied in the food and pharmaceutical industries.^4^ The most abundant bioactive compounds in BSG are hydroxycinnamic acids (HCAs).^5^ These HCAs in BSG have antioxidant activity, which could allow for their application in pharmacology and the food industry. Ferulic acid (FA), caffeic acid (CA), and p-coumaric acid (p- CA) are the types of HCAs that have been reported most frequently in the extraction of phenolic compounds from BSG. They are known for their antioxidant, anti-inflammatory, and anticarcinogenic properties.^6^

Various methods have been used for the extraction of other compounds from BSG, such as hydrothermal autohydrolysis for the extraction of oligosaccharides,^7^ dilute acid hydrolysis for the extraction of hemicellulose sugars,^8,9^ enzymatic hydrolysis for the extraction of hemicellulose and lignin^9,10^ ultrasound-assisted extraction to obtain extracts rich in arabinoxylan,^11^ and microwave-assisted extraction with DES to obtain phenolic acids.^12,13^

Several studies point to deep eutectic solvents (DES) as an alternative to replace conventional organic solvents for a more sustainable extraction process.^14^ DESs are green solvents consisting of mixtures of two or more components, a hydrogen bond acceptor (HBA), and one or more hydrogen bond donors (HBD).^15^ DES is an attractive alternative due to its low or nonexistent toxicity, biodegradability, and recyclability. Some recent studies have already demonstrated that DES can be a good alternative in the recovery of bioactive compounds from brewery spent grains.^16,17^

The DES is called deep because it forms an eutectic with a melting point much lower than that of constituents.^18^ Martins et al.^19^ explained the importance of analyzing the ideality of the mixture to avoid overgeneralization and presented a more rigorous definition of DES. According to Martins et al.,^19^ DESs are not pure compounds but mixtures with hydrogen bonds that have differences between the ideal eutectic point (TE, ideal) and the real eutectic point (TE). The first DES presented only the differences between the melting points of the pure components and the mixture; however, this concept was insufficient to distinguish one DES from another eutectic combination.

In this work, four choline chloride-based DESs were produced and their capacity to extract phenolic compounds was evaluated from BSG. Furthermore, a two-stage ultrasound-assisted process was developed to improve the extraction of phenolics, while avoiding the extraction of sugars and fatty acid esters.

## 2 Experimental Section

### 2.1 Brewer’s Spent Grain

The dried BSG was donated by a local brewery (Fortaleza, Brazil). The BSG was grounded in a knife mill and sieved in an electromagnetic sieve machine. BSG-ground particles with average diameters of 0.250, 0.533, and 1.000 mm were obtained and stored in transparent polyethylene bags. Only particles with an average diameter of 0.250 mm were used in the extraction experiments due to their higher homogeneity, as indicated by scanning electron microscopy (SEM) analysis.

### 2.2 Chemicals

Distilled water purified by a Millipore-Q system, ethanol (Synth, Brazil), hexane (Synth, Brazil), Folin-Ciocalteu reagent, sodium carbonate (99.5–100.5%), choline chloride (≥98%), gallic acid (≥99.0), and glycerol (≥99.5) were purchased from Sigma-Aldrich. Lactic acid (85%), citric acid (P.A), and oxalic acid (P.A) were purchased from Dinâmica Qumica Contemporânea (Indaiatuba, Brazil).

### 2.3 Deep Eutectic Solvent Preparation

Four DESs were prepared. Table S1 presents the details of each DES, their components, molar ratio, temperature, and preparation time. The hydrogen bond acceptor (HBA) for all DESs was choline chloride, while the hydrogen bond donors (HBD) varied between three carboxylic acids and polyalcohol. All DESs were prepared in a molar ratio of 1:2, except for DES ChCl:OA, which remained liquid at room temperature only in a molar ratio of 1:1.^27^ ^,^ ^28^

Table S2 presents the molar mass and mass of the reagents used to produce the four DESs used in this work. The table also presents the final moisture content of the DES.

ChCl:LA, ChCl:OA, and ChCl:Gly were prepared by the stirring and heating method proposed by Van Dai et al.^20^ In this method, HBA and HBD are mixed and heated in a water bath at 60 °C under continuous stirring until a transparent, colorless liquid is formed. ChCl:CA was prepared by the vacuum evaporation method proposed by Van Dai et al.^20^

Aqueous mixtures of DESs were also prepared. For the aqueous solutions of DES, 25% Milli-Q water (v/v) was added to 100 mL of ChCl:LA and ChCl:Gly to analyze the effect of water addition on extraction potential. Aqueous solutions were evaluated because some studies reported that water reduces the viscosity of DES, contributing to a higher mass transfer efficacy and a higher extraction potential.^21,22^ No water was added to ChCl:CA since this DES was already prepared with water and had the highest moisture content. ChCl:OA solidified by adding water, so it was not evaluated in the extractions. All DESs were stored in hermetically sealed glass vials until analysis.

These four DESs were chosen based on previous studies that highlighted their application in the extraction of phenolic compounds from agro-industrial residues and other lignocellulosic materials.^23–25^

### 2.4 DES Characterization

#### 2.4.1 Density and Viscosity

Density (ρ) and viscosity (η) were measured in the temperature range from 20 to 70 °C, using a digital oscillation viscosimeter (Anton Paar SVM 3000 U-Tube). Both measurements were performed simultaneously and duplicated by the equipment itself. The viscosimeter is accurate for up to ±0.005 °C for temperature, ±0.0005 g cm^–3^ for density, and ±0.35% for apparent viscosity.

#### 2.4.2 Determination of the Water Content and Surface Tension

The water content in each DES was estimated by Karl Fisher (Metrohm 870 KF Titrino plus) in a 3:1 chloroform/methanol solution (v/v). The water content values reported are the averages of at least three measurements.

Surface tension was measured using the Du Nouey ring method with a Kruess EasyDine K-20 tensiometer controlled by a thermostatic bath (Julabo model F25-ED) and a Pt-100 temperature sensor. The analyses were performed in triplicate over a temperature range of 30–70 °C. The instrument was calibrated with water and toluene at 20 °C. The equipment uncertainties are 0.01 mN/m for surface tension and 0.1 °C for temperature.

#### 2.4.3 Chemical Characterization

The chemical characterization of DES and its constituents was performed by Fourier transform infrared spectrometry (FTIR) using an Agilent Technologies Cary 630 spectrometer. Transmission spectra in a range of 650–4000 cm^–1^ were acquired with a spectral resolution of 1 cm^–1^. The analysis was carried out to identify the chemical composition of the mixture through its functional groups.

### 2.5 Extraction of Phenolic Compounds

#### 2.5.1 DES Selection Assays

The first assay was carried out to select the best two DESs for the extraction of phenolics from BSG. The extraction process was carried out on a probe ultrasound (Unique model DES500) using a titanium macro tip of 13 mm diameter and frequency of 20 kHz and applying an ultrasonic power density of 8,300 W/L. The mass of the sample-to-solvent ratio was fixed at 1:10 (w/v), so 6 g of BSG was mixed with 60 mL of DES in a 100 mL jacketed Becker. A circulating water bath set at 30 °C was coupled to the jacketed Becker to ensure a constant temperature during the extraction. The extraction was carried out for 10 min. After extraction, the samples were centrifuged at 10,000 rpm for 15 min at 25 °C in a refrigerated centrifuge (Sigma model 2–16KL).

A reference experiment following the same procedure was performed using ethanol as the solvent. This reference experiment was carried out for comparison since ethanol is the main green solvent used in the extraction of phenolic compounds.

#### 2.5.2 Ultrasound-Assisted Extraction

The assays for the selection of the two best DESs for phenolic extraction pointed out that a reasonable amount of sugars, fatty acids, and fatty acid esters were extracted along with the phenolic compounds. Therefore, a strategy for the cleanup of the samples was developed to improve the quality of the phenolic extracts. Furthermore, chemical characterization of the extracts showed that the high ultrasonic power density imposed by the probe ultrasound probably caused the degrading of part of the phenolic compounds.

A two-stage process was implemented to improve the quality of the phenolic extract. The first stage of the process referred to a cleanup stage, where ethanol and hexane were chosen as cleanup solvents to remove most of the sugars, fatty acids, and fatty acid esters from BSG.

ChCl:LA and ChCl:OA were employed as solvents for the second stage, where most of the phenolic compounds would be extracted. The selection of these DESs was informed by preliminary screening experiments described in Section 2.5.1. Both stages were carried out in an ultrasonic bath to deliver a lower ultrasonic power density, avoiding the degradation of phenolic compounds.

In the first stage, a solid/solvent ratio of 1:10 (w/v) was used, and 6 g of BSG was mixed with 60 mL of ethanol or hexane. The cleanup was carried out in an ultrasonic bath at 30 °C for 30 min. The operation was carried out at an ultrasonic power density of 55 W/L. The extracted liquid was removed, and the remaining solids were dried in a circulating oven at 60 °C for 24 h.

In the second stage, the dried solid phase from the first stage was mixed with 60 mL of ChCl:LA or ChCl:OA and subjected to sonication. The extraction was performed in triplicate at 30, 40, and 50 °C. The total extraction time was 30 min, with 2 mL of aliquots taken after 5, 10, 20, and 30 min. The operation was carried out at an ultrasonic power density of 55 W/L.

### 2.6 Total Phenolic Content

Total phenolic content (TPC), expressed in milligrams of gallic acid equivalent per liter of BSG extract (mg GAE L^–1^), was determined according to the Folin–Ciocalteu method with the modifications proposed by Almeida et al.^26^ The procedure consisted of mixing 0.100 mL of sample with 0.200 mL of Folin-Ciocalteu reagent (20%, v:v) and 2.000 mL of distilled water. After 3 min, the reaction was stopped by adding 1.000 mL of Na_2_CO_3_ (20% w/v). A reference sample was produced by mixing 0.100 mL of distilled water with the sample. The absorbance was measured using a UV–vis spectrophotometer (Thermos model Evolution 200) at a wavelength of 765 nm.

### 2.7 Statistical Analysis

All experiments and measurements were made in triplicate. Standard deviations were calculated based on raw data. For statistical data analysis, one-way variance analysis (ANOVA) was performed using Origin software (Origin Lab, 2023 Corporation, USA). The mean values of each factor were compared using Tukey’s test at a probability level of 95%.

## 3 Results and Discussion

### 3.1 BSG Surface Characterization

The surface morphology of the BSG after physical treatments (milling and sieving) was evaluated by using SEM. Figure 1 shows the surface of the BSG particles with an average diameter of 0.250 mm at different augmentations. A heterogeneous distribution of grains was observed, with some broken fibrils of different sizes and thicknesses, possibly originating from grain husks, which generally have a more fibrous character. Some gaps could be observed in the form of pores and rough surfaces with laminar and granular particles.

**Figure 1:**
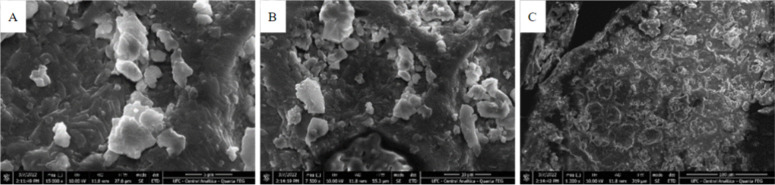
SEM micrographs of BSG particles with an average diameter of 0.250 mm at different augmentations: (a) 15,000×; (b) 7500×, and (c) 1300×.

### 3.2 DES Characterization

#### 3.2.1 Density

The density of the four studied DES was determined as a function of temperature. Figure 2 shows the effect of the increase in the temperature on the decrease in the solvent density. The complete data are listed in Table S3.

**Figure 2:**
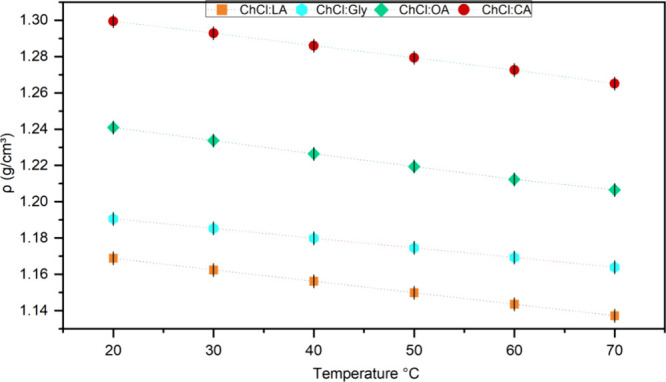
Density of ChCl:LA, ChCl:Gly, ChCl:OA, and ChCl:CA as a function of the temperature.

ChCl:CA exhibited the highest density index, with a maximum of 1.30 g/cm^3^ at 20 °C and a minimum of 1.27 g/cm^3^ at 70 °C. ChCl:OA also exhibited high density, with a maximum of 1.24 g/cm^3^ at 20 °C and a minimum of 1.21 g/cm^3^ at 70 °C. This high density directly affects the performance of the solvent in extraction processes, as it hinders diffusivity and mass transfer.^23^ Another study found similar density values for ChCl:OA.^24^ ChCl:Gly and ChCl:LA showed the lowest density values, with a maximum value of 1.19 and 1.17 g/cm^3^ at 20 °C, respectively, and a minimum value of 1.16 and 1.14 g/cm^3^ at 70 °C. Hence, the density curve can be arranged in the following order: ChCl:LA < ChCl:Gly < ChCl:OA < ChCl:CA, where the last value is the DES with the highest density. Table 1 presents the parameters of the linear equations (*d* = *a* · *T* + *b*) correlating temperature with density, obtained by least-squares analysis. The relationship between density and temperature was extremely linear with an R^2^ value between 0.998 and 1.000 for all DES examined.

**Table 1:** Parameters of the Density Model Together with the Root Mean Square Deviations of the Root

DES	*a*	*B*	RMSD	*R* ^2^
ChCl:LA	–0.0063	1.1752	1.1531	1.0000
ChCl:AO	–0.0069	1.2475	1.2233	0.9988
ChCl:CA	–0.0068	1.3065	1.2826	0.9997
ChCl:Gly	–0.0053	1.1959	1.1773	1.0000

#### 3.2.2 Viscosity

Viscosity is an important property for DESs, as it reflects the flow resistance of the solvent. Figure 3 and Table S4 show the viscosity of the four DESs synthesized in this work. As the temperature increased, the viscosity decreased due to the gradual weakening of the hydrogen bonds during the heating process. ChCl:OA and ChCl:Gly presented higher viscosity values. At 20 and 30 °C, the viscosity of ChCl:OA was higher than that of ChCl:Gly. Above 40 °C, ChCl:Gly became more viscous than ChCl:OA. DES ChCl:CA and ChCl:LA were less viscous and had similar viscosities. ChCl:CA remained more viscous than ChCl:LA at all temperatures, with an average difference of 0.7 mPa s from 30 to 70 °C.

**Figure 3:**
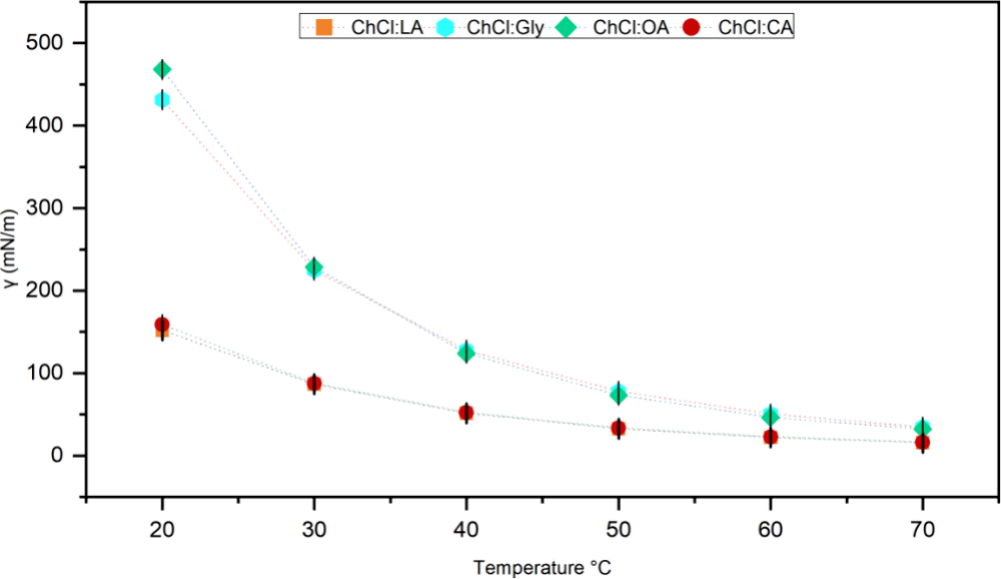
Viscosity of ChCl:LA, ChCl:Gly, ChCl:OA, and ChCl:CA as a function of temperature.

Equally dense substances can exhibit significant variations in the viscosity. This was observed in ChCl:CA, which was the solvent with the highest density (Figure 2) and the second lowest viscosity (Figure 3). As mentioned above, intermolecular forces strongly influence viscosity. ChCl:CA was the only DES prepared with water, which may have weakened hydrogen bonds because the addition of water affects the intra- and intermolecular bonds that are behind the supramolecular network of DES.^29^ The viscosity trend can then be arranged in the following order: ChCl:LA < ChCl:CA < ChCl:Gly < ChCl:OA, the latter being the DES with the highest viscosity.

The dynamic viscosity results were further adjusted by the Arrhenius equation (eq 1). Table 2 presents the parameters for the Arrhenius equation determined from the experimental data and the root-mean-square deviations (RMSD).

$$\eta =\eta_{\infty }e^{E_{a}/R.T}$$ 1

Where η is the dynamic viscosity (mPa·s), η_*∞*_ is the infinite dynamic viscosity (mPa·s), *E*_a_ is the activation energy (kJ·mol^–1^), *R* is the gas constant (kJ·mol^–1^·K^–1^), and *T* is the temperature (K).

**Table 2:** Adjustment Parameters for the Arrhenius Equation for the Dynamic Viscosity of ChCl:LA, ChCl:Gly, ChCl:OA, and ChCl:CA Determined within the Temperature Range *T* = (293.15–343.15) K and *P* = 0.1 MPa

DES	η_∞_ (mPa·s)	*E*_a_ (KJ·mol^–1^)	RMSD	*R* ^2^
ChCl:LA	2.32 × 10^–5^	38.14	3.8734	0.9979
ChCl:AO	4.41 × 10^–5^	44.83	4.7572	0.9952
ChCl:CA	2.37 × 10^–5^	38.16	3.8994	0.9968
ChCl:Gly	1.21 × 10^–5^	42.22	4.7703	0.9974

#### 3.2.3 Superficial Tension

Water absorption is inevitable for some DESs due to their hygroscopic nature.^24^ Synthesized DES, ChCl:OA, ChCl:LA, and ChCl:Gly contained between 2.75 and 8.65% moisture (Table 3). It is important to note that ChCl:CA was the only solvent made with water, which underwent a long rotary evaporation process and had a final moisture content of 8%. While not technically an impurity, the presence of water in DES must be carefully assessed, as it can affect the physicochemical properties of solvents, including density, viscosity, melting point, and surface tension. Evaluating the surface tension of a fluid is crucial in determining its appropriateness for mass transfer processes, determining the energy needed to expand the surface area of the liquid and interpreting the strength of the intermolecular force.^30,31^

**Table 3:** Water Content in DES (%) and Surface Tension in mN/m

		surface tension (mN/m)				
DES	water (%)	30 °C	40 °C	50 °C	60 °C	70 °C
ChCl:LA	3.94	47.1	45.8	45.1	45.0	44.2
ChCl:OA	4.08	66.6	62.1	59.7	58.4	58.3
ChCl:CA	8.65	46.5	45.2	44.5	43.8	43.2
ChCl:Gly	2.75	49.6	49.4	48.5	48.2	47.3

The surface tension tendency showed the following order: ChCl:LA < ChCl:CA < ChCl:Gly < ChCl:OA, the latter being the DES with the highest surface tension. Despite the limited data available in the literature on the surface tension of DES, Chen et al.^32^ reported the surface tension of 50 DESs, two of which have compositions similar to those developed here. The results obtained herein and by Chen et al.^32^ were compared in Table 4. The results obtained for ChCl:LA showed a low variance, while those for ChCl:Gly showed considerable differences, probably because of the amount of water in the mixture. Literature data were not found for DES ChCl:CA and ChCl:OA.

**Table 4:** Comparison of Surface Tension Results Obtained in This Study with Available Data Acquired by Chen et al.^32^

DES	molar ratio (mol mol^–1^)	temperature (°C)	γ this work (mN m^–1^)	γ Chen et al.^32^ (mN m^–1^)	difference (mN m^–1^)
CHCL:LA	1:2	30	47.1	47.1	0
		40	45.8	46.5	–0.7
		50	45.0	45.1	–0.1
		60	44.1	44.1	0
CHCL:GLY	1:2	30	49.6	57.7	–8.1
		40	49.4	57.3	–7.9
		50	48.5	56.7	–8.2
		60	48.2	55.7	–7.5

Other studies showed results similar to those of Chen et al.^32^ for DES diluted in water. For example, Aravena et al.^33^ reported values of γ = 64.9 mN/m at 40 °C, and Chanioti and Tzia^34^ with values of γ = 56.12 mN/m at 40 °C with a diluted DES ChCl:Gly in 20% water (v/v). Chen et al.^32^ did not cite the percentage of moisture content in their mixtures. The water in the medium can reduce or increase the surface tension. Due to the high surface tension of water (72.8 mN/m), diluted DES may have higher values than pure DES. However, adding water in large amounts can also reduce surface tension due to breaking hydrogen bonds.

#### 3.2.4 Chemical Analysis

FTIR spectra of DES and their constituents are presented in Figure S1. The spectra of DES resemble those of their hydrogen bond donors with small absorbance shifts due to mixing with choline chloride. The identification of wave numbers for all DES is shown in Figure 4. The absorbances identified between 3496 and 3200 cm^–1^ indicate the presence of single O–H stretching bonds, which denotes the effective presence of water in the medium.^35^ Stretching vibrations of the DES’s O–H functional group are less intense than those of HBD, indicating that the functional group participates in the formation of the hydrogen bonding interaction between choline chloride anions.^36^ The two weak absorbances corresponding to the C–H stretching bands occurred at 2657–2553 cm^–1^.^37,38^ Low-intensity absorbance at 1725–1613 cm^–1^ may be associated with C=O elongation due to carboxylic acid groups. For ChCl:Gly, the absorbance at 1479 cm^–1^ shows an H flexion, C–C stretching, and other vibrations at 1032 cm^–1^.^39^

**Figure 4:**
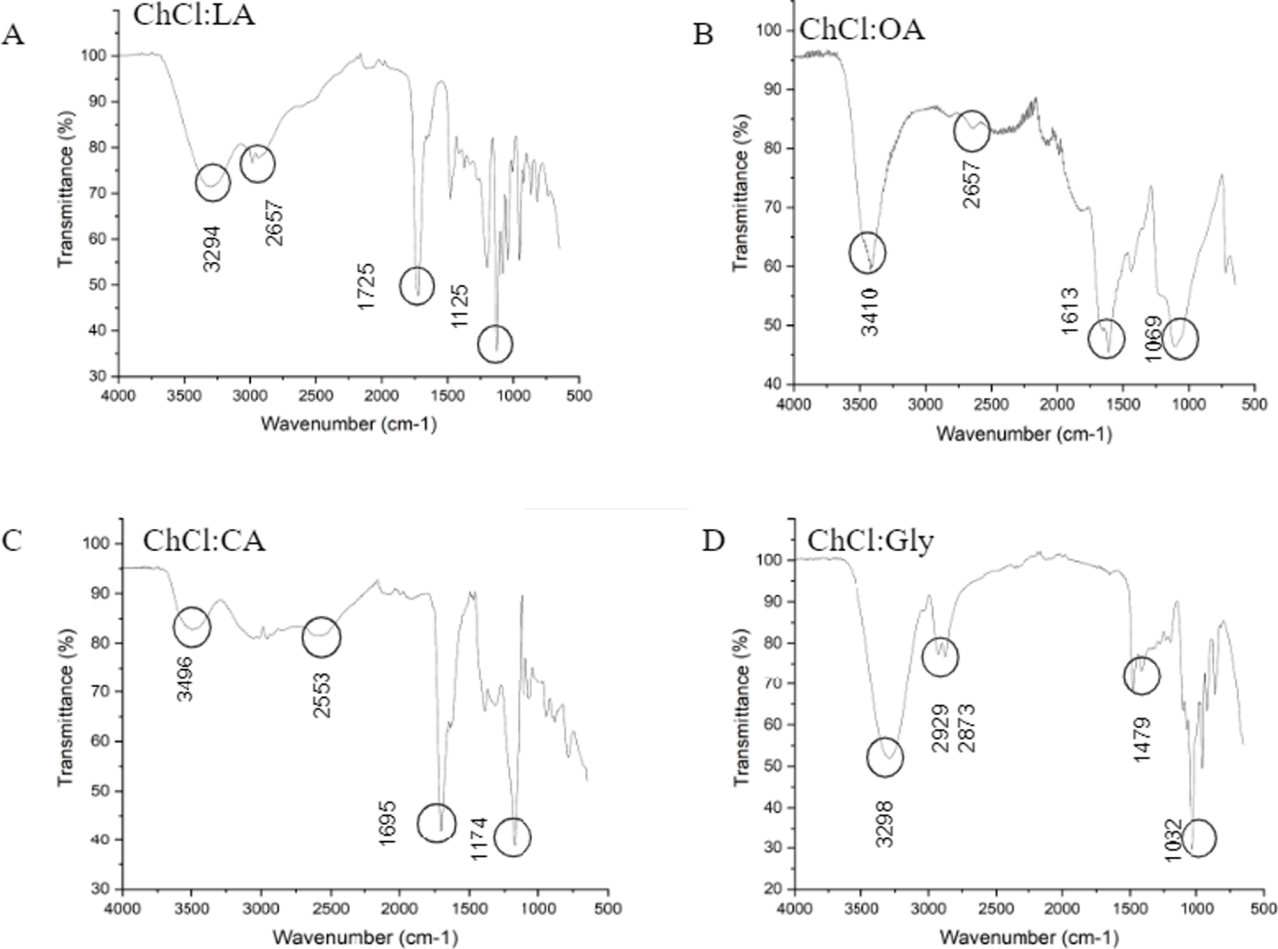
Identification of the FTIR absorbances of (a) ChCl:LA, (b) ChCl:OA, (c) ChCl:CA, and (d) ChCl:Gly.

### 3.3 Extraction and Analysis of Total Phenolics

#### 3.3.1 Selection of the Best DESs for Extraction of Phenolic Compounds

The first set of experiments was performed to narrow the number of DESs for process optimization. From the six initial DESs, the two best-performing DESs were selected for the second set of experiments. Experiments were conducted using high-power, low-frequency ultrasound to evaluate the performance of DES in extracting phenolics and other compounds. The decision of the best performing DES was selected based on the amount of phenolics extracted measured by the Folin–Ciocalteu method. Ethanol was used as the reference solvent for comparison purposes. Figure 5 presents the amount of phenolic compounds extracted from BSG.

**Figure 5:**
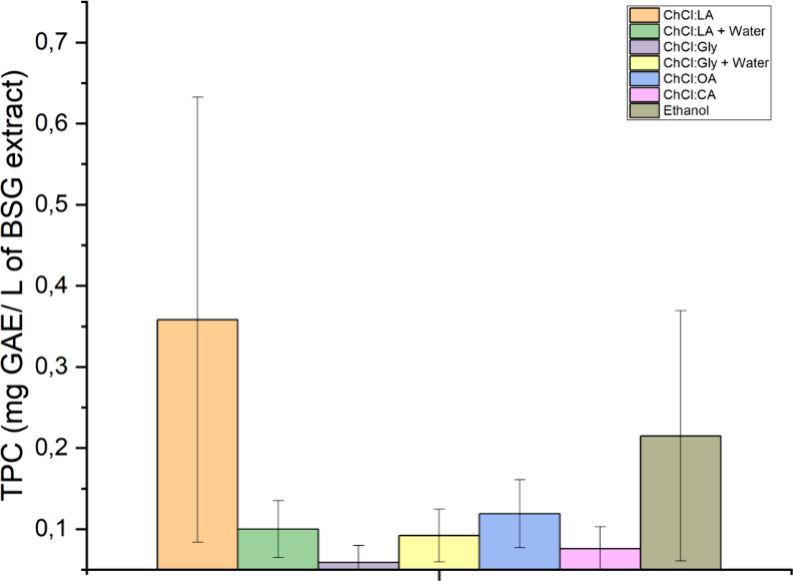
Total phenolic content (mg GAE/L) in BSG extracts obtained from DES and their mixtures with water using ethanol as a reference solvent for comparison.

The TPC ranged from 0.201 to 0.014 mg of GAE/L, following the order ChCl:LA > ethanol > ChCl:OA > (ChCl:LA + water) > (ChCl:Gly + water) > ChCl:CA > ChCl:Gly. The two best-performing DESs were ChCl:LA and ChCl:OA, which were selected for further process optimization. In general, a greater ability to extract phenolic compounds was observed for DES produced from carboxylic acids than from polyalcohols (ChCl:Gly). Our results differed from the results of the study by López-Linares et al.,^23^ in which ChCl:Gly was more efficient in extracting total phenolics from BSG.

The addition of water to ChCl:Gly resulted in a better extraction yield, probably due to its lower viscosity, which improved mass transfer. Other studies also reported that ChCl:Gly added with water was more effective in extracting phenolic compounds than pure ChCl:Gly.^40,41^

The addition of water to ChCl:LA did not have the same effect. The extraction yield decreased by 27% compared to the pure DES. The decrease could be related to an increase in its polarity, which does not favor the extraction of phenolic compounds.^42^

A notable challenge in the TPC analysis was the formation of precipitates during sample preparation using the Folin–Ciocalteu method, which may explain the observed variability in the results shown in Figure 5. Initially, it was hypothesized that DES itself was destabilizing and precipitating when the reagents were added. To rule out this possibility, samples were prepared by mixing DES with each reagent individually and the solutions were monitored for precipitate formation; however, no precipitate was observed. This led to the second hypothesis that precipitation occurred due to the reaction of certain compounds extracted during DES-based extractions. After some literature research, we found that precipitate formation was common in other studies extracting phenolic compounds of agro-industrial materials, and that a cleanup could be a good strategy to remove unwanted products and reduce precipitate formation.^43,44^

The extracts were then evaluated by gas chromatography coupled with mass spectroscopy, which indicated that the extracts contained some phenolics and large amounts of sugars, fatty acids, and fatty acid esters. Thus, a strategy for the cleanup of the samples was shown to be essential to improve the quality of the phenolic extracts. Furthermore, chemical characterization of the extracts showed that the high ultrasonic power density imposed by probe ultrasound probably degraded part of the phenolic compounds, and a lower ultrasonic power density should be employed in the process.

#### 3.3.2 Evaluation of the Two-Stage Process on the Extraction of Phenolic Compounds

A two-stage process was evaluated to improve the quality of the phenolic extract. The first stage of the process is referred to as a cleanup stage, where ethanol and hexane were investigated as cleanup solvents to remove most of the sugars, fatty acids, and fatty acid esters from BSG. Hexane was chosen for its ability to dissolve nonpolar compounds while minimizing the coextraction of phenolic compounds from BSG. Ethanol, on the other hand, was selected for its significantly lower toxicity compared to hexane and other commonly used solvents. Other studies have also used these solvents for the removal of sugars and fatty acids, highlighting their effectiveness in similar applications.^45,46^

ChCl:LA and ChCl:OA were employed as solvents for the second stage, where most of the phenolic compounds would be extracted. Both stages were carried out in an ultrasonic bath to deliver a lower ultrasonic power density avoiding the degradation of phenolic compounds.

Figure 6 presents the TPC of the BSG extracts obtained at 30, 40, and 50 °C. At 30 °C, the most favorable extraction condition using ChCl:LA was achieved after 5 min of sonication using the combination hexane–ChCl:LA, resulting in a TPC of 0.086 mg of GAE L^–1^. The TPC for this system decreased by 88% after 10 min, and no phenolics were detected after 20 min. Although several phenolics are stable under mild sonication (<500 W/L) and near-ambient temperatures (30 °C), the results indicated that the phenolics have degraded during extended sonication. Phenolic compounds were not detected in the extractions carried out with the combination of ethanol-ChCl:LA. In this case, most probably, the phenolics were extracted by ethanol in the cleanup stage, resulting in no further extraction. The hexane–ChCl:OA system at 30 °C also showed the best result at 5 min of sonication, resulting in a TPC of 0.516 mg of GAE L^–1^, which gradually decreased over time. Other studies showed the same relationship with higher extraction efficiency in shorter sonication periods.^47,48^ The ethanol–ChCl:OA system reached the highest efficiency in 20 min, extracting 0.513 mg of GAEL^–1^ of phenolic compounds.

**Figure 6:**
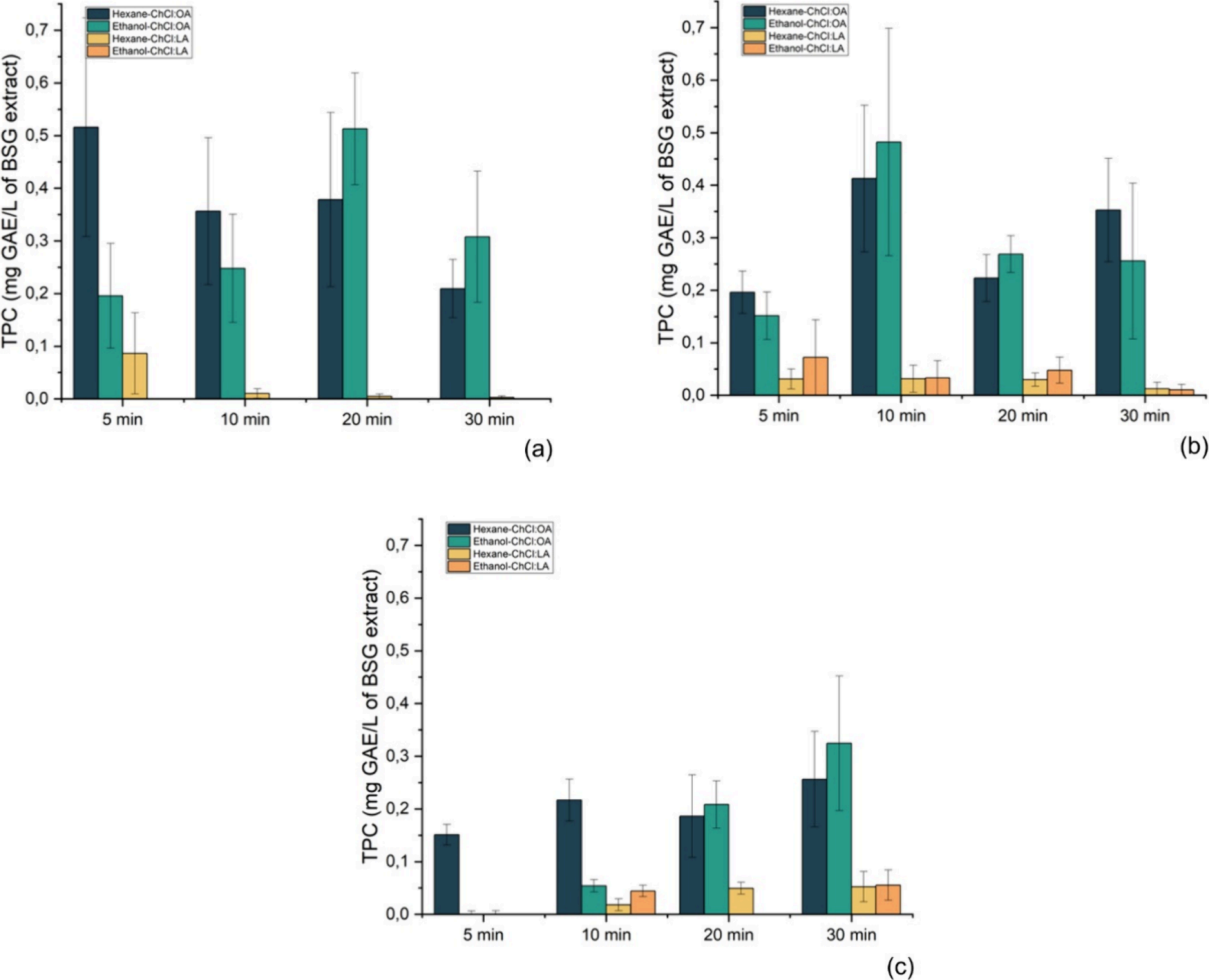
Total phenolic content of Brewers’ Spent Grain after the two-stage extraction process carried out at 30 (a), 40 (b), and 50 °C (c).

At 40 °C, the hexane–ChCl:LA system extracted 0.031 mg of GAE L^–1^ phenolics, a level that was maintained for 20 min, decreasing afterward. It could be concluded that increasing the extraction time did not lead to better results with this system. The best outcome for the combination of ethanol-ChCl:LA was obtained after 5 min with a much better performance (0.072 mg GAE L^–1^) than the hexane–ChCl:LA system. Phenolic compounds concentration decreased after 10 and 20 min, indicating degradation of the phenolics. Once again, ChCl:OA showed a greater capability of extracting phenolics than ChCl:LA. The best extraction condition for the ChCl:OA systems was observed after 10 min of sonication, with 0.413 mg of GAE L^–1^ attained by the hexane-ChCl:OA system and 0.482 mg of GAE L^–1^ obtained with the ethanol–ChCl:OA system. Statistically, there was no difference between the best performance of the hexane–ChCl:OA and the ethanol–ChCl:OA systems at 30 and 40 °C (Table S5).

At 50 °C, the best extraction yields occurred after 30 min of sonication for all systems. As observed with the extractions at 30 and 40 °C, ChCl:LA showed a lower capacity to extract phenolics than ChCl:OA. The ethanol–ChCl:LA system resulted in the extraction of 0.055 mg of GAE L^–1^, while the ethanol–ChCl:OA system resulted in the extraction of 0.325 mg of GAEL^–1^ of phenolic compounds. The biggest difference observed at 50 °C was that the optimal extraction time was 30 min, whereas at 30 and 40 °C, the best results occurred between 5 and 10 min.

When the results obtained at 30 and 40 °C were compared, increasing the temperature did not lead to higher TPC yields. Although higher temperatures favor mass diffusivity, decrease viscosity and surface tension of DES, and improve the release of phenolics from its matrix,^34,49^ these factors did not affect our proposed two-stage process. Higher decomposition rates and loss of stability of phenolic compounds occurred above 40 °C, decreasing the total yield of phenolic compounds in the processes carried out at 50 °C. Serial extraction with hexane as a cleanup solvent was more effective for both DESs, probably because it removed fewer phenolic compounds than ethanol in the first stage.

The inclusion of a cleanup stage was essential to reduce variability in the results. Other studies using DES for extracting various compounds from BSG did not include a cleanup step and relied on higher temperatures (100 and 180 °C) and longer extraction times to achieve satisfactory yields.^23,50^ Consequently, these studies often coextracted residual solid impurities along with the target compounds.

Other studies exploring BSG as a source of valuable compounds highlight the use of ILs as promising solvents for the biorefinery of this agro-industrial waste;^51–53^ however, their application for phenolic compound recovery from BSG remains limited. While this study does not claim superiority over IL-based methods, it demonstrates that DESs, coupled with a cleanup stage, can provide a viable solution for phenolic compound extraction under mild temperatures and shorter extraction times.

In addition to the extraction efficiency demonstrated with the two-stage process (cleanup and extraction), a third stage should be added to enhance the environmental and economic value of the process: the reuse of DES. A comprehensive review by Isci and Kaltschmitt,^54^ encompassing more than 40 studies, highlights the potential for recycling DESs over 4, 5, and even 10 cycles without significantly compromising their extraction efficiency. Although the recycling of DESs was not experimentally evaluated in this study, DESs composed of choline chloride and carboxylic acids have been successfully recovered and reused in other studies.^55–57^ Wang et al.^55^ showed that ChCl:OA can be reused up to 10 times for biomass pretreatment if, after each cycle, the DES is filtered, recovered using a rotary evaporator, and its composition is adjusted with oxalic acid or fresh DES to restore its original composition before the next cycle.

According to Yiin et al.,^58^ the current challenge in the use of DESs is the high cost of the raw chemicals required for their production. However, these costs could potentially be offset by the use of DES in multiproduct biorefineries that focus on high-value, low-volume products rather than conventional low-value, high-volume products.^59^ DESs have the potential to contribute to these advanced biorefineries by enabling the extraction of multiple products by first acting in the removal of sugars and fatty acids and then being recycled and reused to extract other valuable compounds.

Moreover, as demonstrated by the results of this study, DESs not only show great promise in recovering bioactive compounds but also reduce operational costs through a lower energy consumption. The process developed here operates at mild temperatures (30–50 °C) and requires short extraction times, showcasing the potential of DESs to combine environmental and economic benefits in a sustainable extraction process.

#### 3.3.3 Influence of Viscosity on Extraction Efficiency

It is often argued that the relatively high viscosity of DESs is a significant limitation in extraction processes, as the transport rate of molecules from the solid substrate to the solvent is reduced, thus decreasing the efficiency of the extraction process.^60^ Also, in microwave or ultrasound-assisted extraction processes, the high viscosity of DES can reduce cavitation due to the viscous forces limiting propagation of the shockwaves through the liquid.^61^

The viscosity of the DES used in the two-stage extraction made a great difference. DES ChCl:LA had a significantly lower dynamic viscosity of 86.75 mPas at 30 °C than the DES ChCl:OA that demonstrates a higher viscosity of 224.73 mPas at the identical temperature. However, the highest yields of phenolic compounds were obtained with the hexane–ChCl:OA system with the highest TPC during 5 min of sonication at 30 °C as concluded from all combinations tested.

Although the high viscosity of DES ChCl:OA initially raised concerns, its chemical composition may have played a more advantageous role by facilitating deeper penetration into the BSG matrix and enhancing interactions with phenolic compounds. This behavior suggests that the chemical composition of the DES may be as important, if not more so, as its absolute viscosity in extraction processes.

Further investigations are needed to confirm whether, under certain circumstances, a high viscosity could contribute to increased selectivity and improve the quality of phenolic extracts.

### 3.4 Challenges and Perspectives

Despite the promising results in the use of DES for phenol extraction, this study revealed limitations that need to be addressed to enable the industrial application of this process. An interesting finding was that at higher temperatures, the amount of extracted material was reduced. It needs to be investigated whether this reduction is due to the thermal degradation of the phenolic compounds or whether the decrease in DES viscosity also reduces the efficiency of the extraction.

Previously, it was assumed that the viscosity of the DES was only a negative factor in the extraction process, but in this study, the more viscous DES proved to be more effective. Another limitation of the study was the use of the Folin–Ciocalteu method for TPC analysis. Future research should explore complementary analytical methods, such as HPLC or mass spectrometry, to ensure greater consistency and precision in the quantification of the extracted compounds.

To further improve the sustainability of the process, the purification phase could be performed with a DES specifically designed for high selectivity in sugars and oils, while having low efficiency in the extraction of phenolic compounds.

Furthermore, it is crucial to develop strategies to effectively separate DES from the extracted phenolic compounds to ensure their purity and usability in downstream applications

## 4 Conclusions

Four DESs were used to extract phenolic compounds from BSG. A two-stage extraction process was performed due to the need to remove sugars, fatty acids, and fatty acid esters from BSG. Ethanol and hexane were used as the first cleanup solvents. Hexane was the best cleanup solvent at 30 °C, and no statistical difference was observed between the two cleanup solvents at higher temperatures (40 and 50 °C). ChCl:OA was the best-performing DES in the extraction of outstanding phenolic compounds ChCl:LA. The performances of the hexane-ChCl:OA and ethanol-ChCL:OA systems were statistically similar. However, the ethanol–ChCl:OA system required longer sonication periods in comparison to the hexane–ChCl:OA system. DES mixtures with 25% (v/v) water did not yield higher TPC yields. This study concluded that BSG is a residue with satisfactory recycling potential and can be used as a raw material for the synthesis of phenolic compounds.

## Supplementary Material



## Notes

Information on the operating conditions used to produce the deep eutectic solvents (DESs) from choline chloride as the hydrogen bond acceptor and several compounds as hydrogen bond donors, the molar mass and the mass of HBA and HBD used to produce each DES and the final moisture content of the DES, experimental densities and viscosities of DES in the temperature range *T* = (20–70) °C, results of total phenolics obtained in each extraction system (mean and standard deviation), and FTIR of the four DES and their constituents: (a) ChCl:LA, (b) ChCl:OA, (c) ChCl:CA, and (d) ChCl:Gly (PDF)
