# Supplementary material for: Valorization of Brewer’s Spent Grain Through Ultrasound-Assisted Extraction of Phenolic Compounds Using Deep Eutectic Solvents
Source: ACS Omega. 2025 May 29;10(22):22603–12. doi: 10.1021/acsomega.4c10526 (PMC12163671; doi:10.1021/acsomega.4c10526)

Supporting information

Valorization of brewer's spent grain through ultrasound-assisted extraction of phenolic compounds using deep eutectic solvents

Paloma Paiva Santiago[1], Fabiano André Narciso Fernandes[2], Rílvia Saraiva de Santiago-Aguiar [2,*]

[1]Energy, Environmental & Chemical Engineering (EECE), McKelvey School of Engineering, Washington University in St. Louis (WashU), St. Louis, MO 63130-4899, USA.

[2]Federal University of Ceara, Chemical Engineering Department, Campus do Pici, Bloco 709, 60440-900 Fortaleza – CE, Brazil

[*]Corresponding author: Rilvia S. S. Aguiar. Federal University of Ceara, Chemical Engineering Department, Campus do Pici, Bloco 709, 60440-900 Fortaleza – CE, Brazil. Email: rilvia@ufc.br

Table S1. Information on the operating conditions used to produce the Deep Eutectic Solvents (DES) from choline chloride as the hydrogen bond acceptor (HBA) and several compounds as hydrogen bond donors (HBD).

DES	HBA	HBD	Molar Ratio (mol/mol)	Temperature (ºC)	Processing Time (min)
ChCl:LA		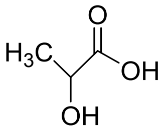 Lactic acid	1:2	60	30
ChCl:CA*		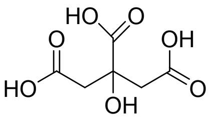 Citric acid	1:2	60	270
ChCl:OA**	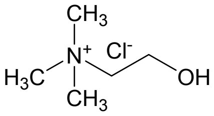 Choline chloride	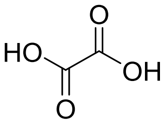 Oxalic Acid	1:1	80	60
ChCl:Gly		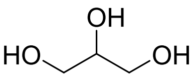 Glycerol	1:2	60 ºC	30

* DES ChCl:CA was synthesized with 50% MilliQ water (w/w).

** DES ChCl:OA only remained liquid at room temperature in a 1:1 molar ratio.

Table S2. The molar mass and the mass of HBA and HBD used to produce each DES and the final moisture content of the DES.

DES		HBA			HBD		
	Moisture (%)		Molar Mass (g/mol)	Mass (g)		Molar Mass (g/mol)	Mass (g)
ChCl:LA	3.94	Choline chloride	139.63	228.06	Lactic acid	90.08	293.66
ChCl:CA	8.65	Choline chloride	139.63	145.52	Citric acid	192.12	399.61
ChCl:OA	4.08	Choline chloride	139.63	407.71	Oxalic Acid	126.07	365.12
ChCl:Gly	2.75	Choline chloride	139.63	279.26	Glycerol	92.09	368.32

Table S3 - Experimental densities of deep eutectic solvents (DES) in the temperature range T = (20 to 70) °C [a]

T / ºC	ChCl:LA	ChCl:Gly	ChCl:OA	ChCl:CA
20	1.1689	1.1906	1.2409	1.2994
30	1.1624	1.1853	1.2337	1.2929
40	1.1562	1.1799	1.2264	1.2860
50	1.1499	1.1746	1.2194	1.2793
60	1.1435	1.1693	1.2123	1.2725
70	1.1372	1.1639	1.2065	1.2651

[a] Standard uncertainties are (T) ± 0.005 °C, (ρ) ± 0.0005 $g.cm^{-3}$

Table S4 - Experimental viscosities of deep eutectic solvents (DES) in the temperature range T = (20 to 70) °C [a]

T / ºC	ChCl:LA	ChCl:Gly	ChCl:OA	ChCl:CA
20	151.970	431.130	468.040	158.890
30	86.748	224.730	228.500	87.687
40	51.458	127.780	123.900	52.415
50	32.768	77.953	73.326	33.539
60	22.119	50.506	46.561	22.723
70	15.669	34.452	32.200	16.217

[a] Standard uncertainties are (T) ± 0.005 °C, (ρ) ± 0.001 mPa.s

Table S5 - Results of total phenolics obtained in each extraction system (mean and standard deviation)

**Extraction yield in TPC (mean ± SD) / (mg GAE. L $^{-1}$)**				
Extraction System	Ethanol-ChCl:LA	Hexane-ChCl:LA	Ethanol-ChCl:OA	Hexane-ChCl:OA
5 min 30 °C	0.000	0.086 ± 0.133	0.196 ± 0.172	0.516 ± 0.359
10 min 30 °C	0.000	0.010 ± 0.015	0.248 ± 0.178	0.356 ± 0.241
20 min 30 °C	0.000	0.005 ± 0.008	0.513 ± 0.184	0.378 ± 0.286
30 min 30 °C	0.000	0.003 ± 0.004	0.308 ± 0.215	0.209 ± 0.096
5 min 40 °C	0.072 ± 0.124	0.031 ± 0.033	0.151 ± 0.078	0.196 ± 0.070
10 min 40 °C	0.033 ± 0.057	0.031 ± 0.045	0.482 ± 0.375	0.413 ± 0.242
20 min 40 °C	0.048 ± 0.042	0.030 ± 0.022	0.269 ± 0.061	0.223 ± 0.078
30 min 40 °C	0.010 ± 0.017	0.012 ± 0.021	0.256 ± 0.257	0.353 ± 0.170
5 min 50 °C	0.000	0.007 ± 0.012	0.006 ± 0.011	0.151 ± 0.036
10 min 50 °C	0.044 ± 0.018	0.018 ± 0.019	0.054 ± 0.020	0.217 ± 0.070
20 min 50 °C	0.000	0.049 ± 0.019	0.208 ± 0.078	0.186 ± 0.136
30 min 50 °C	0.055 ±0.049	0.052 ± 0.049	0.325 ± 0.221	0.256 ± 0.156

Figure S1 - FTIR of the four DES and their constituents: a) ChCl:LA b) ChCl:OA c) ChCl:CA d) ChCl:Gly

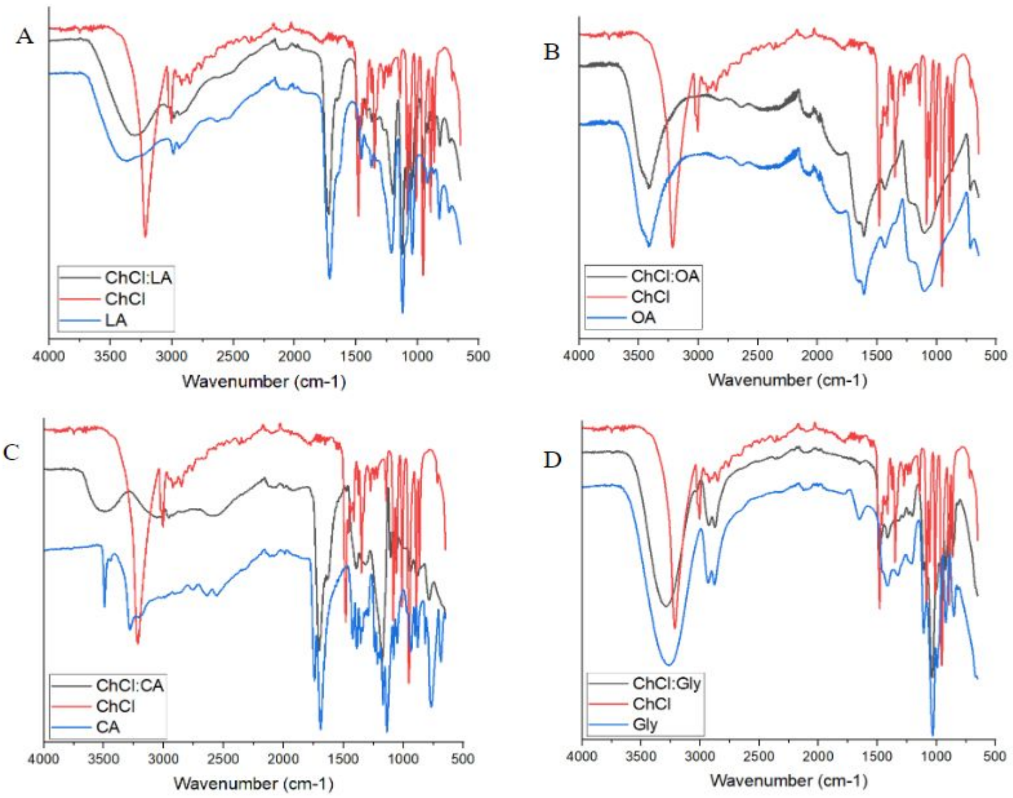

Supplement: Supplementary file 1 [file ao4c10526_si_001.pdf]
